# Long-Term Functional Outcomes of Retinal Detachment Due to Acute Retinal Necrosis: A Case Series

**DOI:** 10.3390/biomedicines12102320

**Published:** 2024-10-11

**Authors:** Ludovico Iannetti, Giacomo Visioli, Ludovico Alisi, Marta Armentano, Maria Pia Pirraglia, Massimo Accorinti, Valerio Di Martino, Magda Gharbiya

**Affiliations:** 1 Ophthalmology Unit, Department of Head and Neck, Policlinico Umberto I University Hospital, Sapienza University of Rome, 00185 Rome, Italy; ludovicoiannetti@gmail.com (L.I.); m.pirraglia@policlinicoumberto1.it (M.P.P.); massimo.accorinti@tiscali.it (M.A.); magda.gharbiya@uniroma1.it (M.G.); 2 Department of Sense Organs, Sapienza University of Rome, Viale del Policlinico 155, 00161 Roma, Italy; giacomo.visioli@uniroma1.it (G.V.); ludovico.alisi@uniroma1.it (L.A.); valerio.dimartino@uniroma1.it (V.D.M.)

**Keywords:** acute retinal necrosis, retinal detachment, uveitis, viral retinitis, pars plana vitrectomy, encircling silicone band, herpes viruses

## Abstract

**Objectives**: To evaluate the long-term anatomical and functional prognosis of patients with retinal detachment (RD) secondary to acute retinal necrosis (ARN) treated with pars plana vitrectomy (PPV). **Methods**: This retrospective case series included 21 eyes from 21 patients with RD secondary to ARN. The study analyzed vitreous or aqueous biopsy results, the impact of antiviral therapy, time to retinal detachment, changes in visual acuity (VA), and anatomical and surgical outcomes. All cases underwent 23-gauge PPV with silicone oil tamponade, and an episcleral encircling band was used in 11 cases. All patients received systemic antiviral therapy at diagnosis. **Results**: Retinal reattachment was achieved in 91% of cases during follow-up, with an average follow-up period of 39.5 ± 36.8 months. The average time from ARN diagnosis to RD onset was 33.3 ± 27.5 days. VZV was detected in 10 eyes through PCR analysis. Significant differences in visual prognosis were found between macula-off and macula-on RD (*p* = 0.048). Eyes with optic nerve head inflammation had worse final VA (*p* = 0.010). No significant difference was observed between preoperative VA and VA at the end of follow-up (*p* = 0.665). **Conclusions**: VZV was the primary virus associated with ARN-related RD. Early involvement of the macula and optic nerve in retinitis negatively impacted the final visual prognosis.

## 1. Introduction

**Figure 1:**
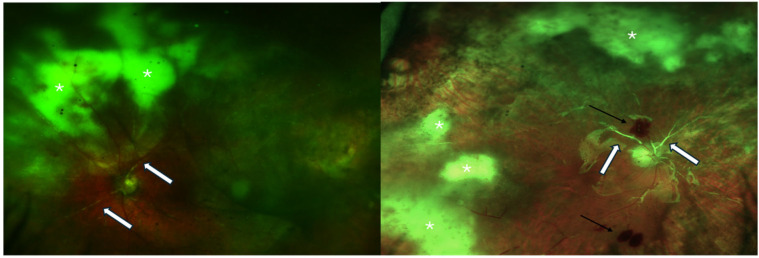
Clinical features of eyes affected by acute retinal necrosis before (**right**) and after (**left**) PPV with silicone oil. White arrows: occlusive vasculitis; white asterisks: areas of confluent retinal necrosis; black arrows: retinal hemorrhages; in both cases, the optic nerve appears involved.

Acute retinal necrosis (ARN) was described for the first time by Urayama et al. in 1971 [1]. It is primarily characterized by progressive peripheral necrotizing retinitis, associated with progressive occlusive vasculitis, mild signs of anterior uveitis, vitritis, and papillitis (Figure 1).

The clinical aspects can be atypical, particularly in the early stages of the disease, and they may not be sufficiently clear to formulate a correct diagnosis [2]. According to the literature [3,4,5,6], in 15–30% of cases, a bilateral ARN occurs, otherwise known as BARN [7]. The incidence of ARN is about 0.63 cases/million per year and can affect individuals of either gender. The diagnosis of ARN is outlined by the American Uveitis Society, whose clinical criteria include: well-demarcated focal areas of retinal necrosis located in the peripheral retina; rapid circumferential progression of necrosis; signs of occlusive vasculitis; and prominent inflammatory response in the vitreous and the anterior chamber [8]. In 50% of cases, the etiology of ARN in immunocompetent individuals is due to varicella zoster virus (VZV) and in 25% to herpes simplex virus (HSV), with occasional cases attributed to Epstein–Barr virus (EBV) and cytomegalovirus (CMV), the latter more commonly causing retinitis in immunocompromised individuals. Although ARN was previously distinguished from progressive outer retinal necrosis (PORN), which primarily affects immunocompromised patients, both conditions are now grouped under necrotizing herpetic retinopathy (NHR), a broader category that includes acute retinal necrosis caused by various herpes viruses [9,10]. The diagnostic criteria for the diagnosis of ARN were first written in 1994 by the American Uveitis Society and subsequently expanded and updated over time [8,9,11]. The polymerase chain reaction (PCR) still represents the gold-standard test for the ARN diagnosis with a specificity of 97% and a sensitivity of 95% [12,13,14]. The medical treatment for ARN consists of an induction phase with systemic antiviral therapy. This is typically administered intravenously—such as acyclovir 10 mg/kg three times daily for 7–10 days—or orally with valacyclovir 2000 mg three times daily until complete resolution. The following maintenance phase involves oral antiviral drugs administered for at least 6 months with a gradual tapering of the dose: acyclovir 800 mg five times daily, valacyclovir 1 g three times daily, or famciclovir 500 mg three times daily. Currently, there is no uniform consensus on which antiviral is a superior option for each pathogen [14,15,16,17].

**Figure 2:**
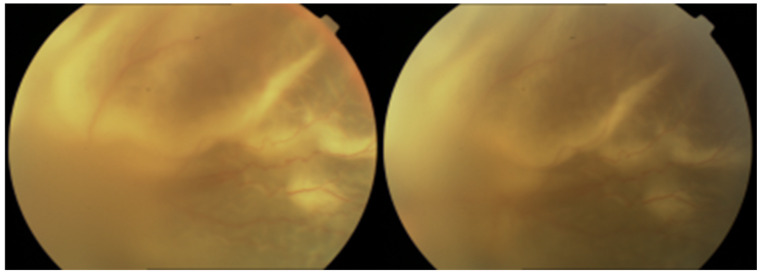
Rhegmatogenous retinal detachment (RRD) in ARN.

In addition to systemic therapy, combining it with intravitreal, antiviral injections has been shown to provide good outcomes in ARN patients. Intravitreal injections of antivirals such as ganciclovir or foscarnet have been successfully used for over 20 years to accelerate the resolution of retinitis and reduce the risk of rhegmatogenous retinal detachment (RRD). This combined approach enhances antiviral effectiveness within the eye, leading to improved patient outcomes [16,17,18,19]. RRD is the major complication of ARN, occurring in 25–75% of cases [20] (Figure 2).

Generally, the RD follows the acute phase of the infection, and it is secondary to vitreous tractions in the areas of retinal necrosis. No difference in RD characteristics has been identified based on the viral etiology, but poor initial VA and the extension of retinitis are strongly associated with this complication [21]. The role played by a retinal photocoagulation laser in preventing RD remains controversial, mainly because it can be applied only if the media remains sufficiently clear [22,23]. Surgery for retinal detachment secondary to ARN has a poor visual prognosis. The literature suggests that prophylactic vitrectomy can reduce the incidence of RD, but its role in BCVA improvement remains controversial [24,25,26,27]. The significant vitreoretinal traction and the high incidence of proliferative vitreoretinopathy (PVR) represent the main causes of surgical failure. For RD secondary to ARN, several authors suggest pars plana vitrectomy surgery, laser endophotocoagulation, and long-term intravitreal tamponade agents (silicone oil) [28,29]. However, it is not possible to outline a uniform, standardized strategy, given the limited number of studies on the subject and the small sample sizes of the patients studied. This research aims to evaluate the long-term anatomical and functional prognosis of patients affected with RD secondary to ARN, who underwent pars plana vitrectomy (PPV), with particular interest in the clinical characteristics and the medical and surgical treatments.

## 2. Materials and Methods

A retrospective study was carried out on a group of patients with RD secondary to ARN followed by the Ocular Immunovirology Service of Policlinico Umberto I, “Sapienza” University of Rome, between January 2020 and December 2023. All patients were diagnosed with ARN, based on medical history and the fundoscopy of retinal necrosis involving peripheral and extending towards the posterior pole areas with a mild hemorrhagic component and/or signs of progressive occlusive vasculitis. All patients, regardless of the stage of the retinal infection, underwent a complete workup for uveitis to identify the etiology of the uveal inflammation and, especially, to exclude any other possible uveal entity. To avoid selection bias, we excluded immunosuppressed patients at the time of diagnosis and those with signs more suggestive of PORN, such as limited intraocular inflammation and predominant involvement of the outer retina. Patients with bilateral ARN were randomized to select only 1 eye to avoid inter-eye correlation.

For every patient, the following data were considered: age of onset, sex, follow-up, viral etiological diagnosis via PCR analysis of aqueous humor and vitreous, possibly associated symmetrical diseases and immune status, medical treatment received, initial and final best corrected visual acuity (BVCA) in LogMAR, time elapsed between diagnosis of ARN and onset of RD, macular involvement in the RD, optic nerve involvement as papillary edema, surgery performed, preoperative lens status (phakic/pseudophakic), and initial and final intraocular pressure with eventual hypotonic treatment.

As far as the type of surgery, a 23-gauge PPV was performed, and the 1000 cSt (centistokes) silicone oil was injected at the end of every procedure. Cataract surgery was concomitantly performed in phakic eyes. All surgeries were performed by the same experienced vitreoretinal surgeon.

Continuous variables, presented as mean ± standard deviation (SD), were assessed for normality using the Shapiro–Wilk test. The Mann–Whitney test was applied for comparisons of non-parametric values, while a paired or unpaired *t*-test was used for parametric data. Categorical variables were expressed as counts and percentages. Statistical analysis was performed using JASP Team (2024), JASP (Version 0.19.0) software.

## 3. Results

A total of 21 eyes of 21 patients (14 men and 7 women) with an average age of 58.8 ± 17.7 (range 26–80) were included in the study. All patients were immunocompetent at the time of diagnosis. Clinical and demographic characteristics are summarized in Table 1. Of note, 2 patients had a past medical history of herpes viral encephalitis; 1 patient had a history of breast cancer, a thyroid nodule, and cutaneous herpes zoster. The average follow-up was 39.5 ± 36.8 months (range of 4–132). The mean initial BCVA was 1.59 ± 0.85 LogMAR, while the final BCVA was 1.52 ± 0.69 LogMAR, with no statistically significant difference (*p* = 0.665). The average time elapsed between the ARN diagnosis, and the RD onset amounted to 33.3 ± 27.5 days. The initial mean intraocular pressure was 13.2 ± 2.7, while the final was 12.5 ± 4.6.

From the PCR analysis, the presence of VZV was the most common, being detected in 10 eyes (9 in the aqueous humor, 1 in the vitreous, and 3 in both intraocular fluids), followed by HZV1/2 detected in 8 eyes. Furthermore, in 2 eyes, there was the presence of HSV1/2 both in the aqueous and in the vitreous, and in 1 eye, there was a simultaneous presence of CMV and HSV1 in the vitreous. Lastly, 2 patients showed CMV positivity (Table 2).

At the time of diagnosis, all patients had undergone systemic antiviral therapy: 3 patients (14.2%) underwent oral therapy with acyclovir 850 mg 5/die; 12 patients (57.1%) received acyclovir 10 mg/kg t.i.d. i.v. as induction treatment; 1 patient was treated with valacyclovir 1 g t.i.d. per os, 2 patients received valacyclovir 1 g b.i.d. i.v. The other 3 patients received mixed therapies during their admittance into other hospital clinics with valacyclovir, ganciclovir, acyclovir, and foscarnet.

In 15 eyes (71.4%), the RD was also present in the macular region (macula-off). In 14 eyes (66.7%), an involvement of the optic disc as papilledema was found. During the RD diagnosis, all the eyes resulted in phakic status. In 11 eyes (52.4%), an encircling band was added during the vitrectomy. In 11 cases (52.3% of eyes), a phacoemulsification with intraocular lens implantation (IOL) in the capsular bag was performed. During the follow-up, 5 eyes (23.8%) underwent phacoemulsification, 3 in a subsequent surgery. In 9 (42.9%), it was decided to remove the silicone oil from the vitreous chamber, while in the rest of the cases, the silicone oil was kept in permanently. In 2 eyes (9.5%), further surgery was required with the replacement of silicone oil due to a relapse onset of RD (Table 3).

The statistical analysis showed a significant difference in terms of BCVA between macula-off RD and macula-on RD at both time points, with a worse BCVA in the macula-off group (*p* = 0.006 at diagnosis and *p* = 0.048 at the end of follow-up). The final BCVA was significantly lower in patients with an initial inflammatory involvement of the optic nerve head. Indeed, the group with optic nerve involvement had the worst BCVA, with preoperative values of 1.91 ± 0.80 LogMAR and postoperative values of 1.83 ± 0.55 LogMAR. These results were significantly worse compared to the group without optic nerve involvement (*p* = 0.001 and *p* = 0.010, respectively). (Table 4).

## 4. Discussion

In this case series, with a substantial long-term follow-up, PPV was effective in achieving good anatomical outcomes in patients with RD due to ARN. However, the final visual acuity was largely determined by the baseline BCVA. Additionally, eyes with macula-off or optic nerve head involvement exhibited poorer visual outcomes.

The ARN is based on viral etiology and can be caused by HSV-1 and HSV-2, VZV, and rarely, CMV or EBV [30,31,32,33]. Different methodologies were used to confirm the viral etiology of ARN, such as retinal biopsies, immunocytochemical analysis, viral cultures, antibody production in the aqueous humor, and most recently, PCR technique [12,14]. Our PCR results showed that the most frequent cause of ARN is VZV. Our results appear in line with other studies conducted in different countries [8,26,34]. No substantial geographic differences appear in the current literature on etiological causes for ARN [25,35,36].

Moreover, it is known that CMV is the main cause of retinitis in immunocompromised individuals, presenting with distinct clinical characteristics that differ from ARN and fall under the broader category of NHR. Despite this, the cases of ARN caused by CMV were reported in not severely immunocompromised patients [37]. In our study, only 2 cases presented CMV with 1 testing positive also for HSV-1. In particular, that single patient had a past medical history of herpes viral encephalitis. In the past literature, a similar background of immune dysregulation in subjects with ARN has been underlined, despite being immunocompetent [38]. It was proven that the central nervous system was involved in herpes viral encephalitis in about 20% of patients with ARN, and our results confirm such an observation [39], with 2 patients presenting with this condition. Therefore, it is recommended to pay particular attention to patients with a medical history of herpes viral encephalitis showing ocular symptoms, due to the high risk of developing a retinal infection.

For all cases, we initiated systemic antiviral therapy at the time of diagnosis. The traditional ARN treatment consists of antivirals i.v. during the induction phase followed by oral maintenance therapy. However, recent studies have shown that oral antiviral therapy is effective even during the initial phases of the disease, resulting in a complete regression of retinitis [40,41]. Interestingly, a recent meta-analysis found no significant differences in final BCVA outcomes based on the chosen treatment regimen. Specifically, it revealed no notable BCVA differences between patients treated with intravenous, oral antivirals, or a combination of systemic and intravitreal antivirals. The authors were not able to demonstrate a superior visual outcome comparing the different antiviral molecules. Care should be taken due to the rising amount of HSV acyclovir-resistant strains, while VZV-resistant strains appear to be far less common [19].

In the examined group of patients, the visual prognosis was poor, despite a final postoperative anatomic success in all cases, in accordance with the literature [34,42,43]. Nonetheless, it is important to notice how the final visual prognosis (final BCVA 1.52 ± 0.69 LogMAR) depends mainly on an initial poor BCVA at the time of diagnosis (initial BCVA 1.59 ± 0.85 LogMAR). The data of our study show how at the end of the follow-up period the initial VA remains substantially unchanged. This suggests that the final visual prognosis is primarily dependent on the baseline BCVA, which in turn, is likely influenced by the extent of macular involvement and optic nerve head alterations. In agreement with our findings, other studies have also shown similar results. Iwahashi-Shima et al. conducted a study on 104 patients affected with ARN with more than 1 year of follow-up to analyze the main factors influencing the visual and anatomical outcomes. They found that a poor final visual prognosis was associated with VZV infection, severe visual loss at presentation, and optic disc involvement. The authors moreover suggested that optic nerve involvement results in an augmented risk for DR development [25]. The involvement of the optic nerve in the initial phase of the ARN was widely reported and can manifest itself as edema of the optic nerve head and papillitis in the initial phase of the disease, followed by a papillary pallor sign of optic atrophy/sub-atrophy of ischemic origin. Cases in which the optic disk appears normal during the ophthalmoscopic examination were reported, but damage secondary to optic retrobulbar neuritis arose simultaneously with ARN in patients with AIDS [44,45,46,47]. Consistently with other authors, the ischemic damage of the optic nerve caused by occlusive vasculitis seems to be the main cause of the poor visual prognosis rather than the RD itself [21]. In our study, we found an involvement of the optical nerve in 66.7% of the cases. The data vary between 50% and 60% of the cases, depending on the authors [48]. Several mechanisms were proposed to explain the link between ARN and optical neuropathy, such as intraneural vasculitis, the presence of exudates located in the optic nerve sheath causing ischemic compression, inflammation, and necrosis directly due to a herpes virus infection [48]. In the literature, other authors showed that the causes with an impact on final BCVA are initial visual acuity, RD complications (i.e., epiretinal membrane formation), treatment delay, localization, and diffusion of the retinal lesions [42,43,49,50,51]. In our patients, the average time elapsed between the ARN diagnosis and the RD onset was about 33 days, following the results of Tibbetts et al. [52]. The surgical technique we used is the same as that suggested by other authors [29]. The final anatomic success with retinal reattachment was obtained in 90.5% of cases. In 2 cases, a reoperation with a replacement of silicone oil due to a relapse onset of RD due to proliferative vitreoretinopathy (PVR) was required. In 42.9% of cases, the silicone oil was removed; in the remaining cases, it was left in place permanently. The decision to retain the silicone oil permanently is based on its ability to effectively stabilize the necrotic retina. Additionally, removing the oil does not lead to significant visual improvement in eyes that already have compromised visual acuity. Moharana et al. performed a 25-gauge micro-incision vitrectomy surgery with silicone oil filling, combined with an encircling band, in 14 ARN eyes with RRD involving the macular area. They reported good functional success in 6 patients who showed a post-surgical improvement of the BCVA. Poor functional outcomes were attributed to the presence of ischemic optic atrophy [53]. The role of prophylactic vitrectomy in ARN-affected patients is controversial, as is retinal laser therapy [54]. Some authors reported an improvement in the visual prognosis in patients who underwent prophylactic vitrectomy, while others consider such methodology irrelevant or even detrimental [26]. Liu et al. demonstrated that prophylactic vitrectomy is effective in preventing retinal detachment in patients with ARN; however, it does not prevent deterioration of visual acuity, mainly because it cannot prevent PVR. The authors suggest that long-term macular damage caused by oil tamponade or other chronic complications may also be responsible for visual deterioration [55]. A recent meta-analysis concluded that prophylactic vitrectomy prevents RRD in ARN patients without any significant improvement in BCVA when compared to medically treated patients [56].

In conclusion, while the role of prophylactic PPV is still debated, for patients presented with RRD determined by ARN, prompt surgery is the most preferable approach to reach a complete anatomic success and to prevent the extension of the disease to the posterior pole, potentially determining macular ischemia or optic atrophy, especially if we take into consideration the high risk of rapidly developing PVR in ARN affected eyes due to the stimulation induced by inflammatory mediators. The mean follow-up of our study is considerable (39.5 months), especially if compared to other studies on the topic. This could contribute valuable insights to the literature, given the good anatomic outcomes observed, even with a relatively small sample size, which is due to the low incidence of the disease.

## 5. Conclusions

This research evaluated the long-term visual and anatomic prognosis of patients with RD secondary to ARN who underwent PPV. The VZV was found to be the main responsible virus for ARN associated with RD, with an interval of around 30 days between the development of symptoms and RD. Optic nerve and macular involvement, likely due to greater severity of necrosis in the early stages of retinitis, negatively affects the final visual prognosis. Although vitrectomy yields excellent anatomic results, relapses—primarily due to the onset of PVR—can occur, and the medium- to long-term visual prognosis is generally poor.

## Figures and Tables

**Table 1:** Demographic and clinical characteristics of the studied population. BCVA: best corrected visual acuity; F: female; IOP: intraocular pressure; RD: retinal detachment.

	Patients (N = 21)
Age	58.8 ± 17.7
Sex (F)	7 (33.3%)
Immunocompetence	21 (100%)
Follow-up (months)	39.5 ± 36.8
Time to RD (days)	33.3 ± 27.5
BCVA (LogMAR pre)	1.59 ± 0.85
BCVA (LogMAR end of follow-up)	1.52 ± 0.69
IOP pre (mmHg)	13.2 ± 2.7
IOP post (mmHg)	12.5 ± 4.6
Comorbidities	
Diabetes	4 (19.0%)
Herpetic encephalitis	2 (9.5%)
Hypothyroidism	2 (9.5%)
Hypertension	7 (33.3%)

**Table 2:** PCR diagnosis on aqueous and vitreous. CMV: cytomegalovirus; EBV: Epstein–Barr virus; HSV: herpes simplex virus; PCR: polymerase chain reaction; VZV: varicella zoster virus.

PCR Diagnosis			
Patients (N = 21)	Aqueous	Vitreous	Both
VZV	9 (40.9%)	1 (4.7%)	3 (14.2%)
HSV1/2	7 (33.3%)	1 (4,7%)	2 (9.5%)
EBV	2 (9.5%)	0	0
CMV	1 (4.7%)	1 (4.7%)	0

**Table 3:** Surgical characteristics of the studied population. EB: encircling silicone band; ON: optic nerve; RD: retinal detachment; SO: silicone oil.

	Yes	No
ON involvement	14 (66.7%)	7 (33.3%)
EB	11 (52.4%)	10 (47.6%)
SO removal	9 (42.9%)	12 (57.1%)
RD recrudescence	2 (9.5%)	19 (90.5%)
	On	Off
Macular status	6 (28.6%)	15 (71.4%)
	Phakic	Pseudophakic
Lens status	19 (90.5%)	2 (9.5%)

**Table 4:** Comparison of BCVA results stratified for clinical characteristics of the studied population. BCVA: best corrected visual acuity; ON: optic nerve involvement.

	Macula Off (N = 15)	Macula On (N = 6)
BCVA (LogMAR pre)	1.89 ± 0.77	0.83 ± 0.50
BCVA (LogMAR end of follow-up)	1.70 ± 0.68	1.05 ± 0.51
	ON yes (N = 14)	ON no (N = 7)
BCVA (LogMAR pre)	1.91 ± 0.80	0.94 ± 0.52
BCVA (LogMAR end of follow-up)	1.83 ± 0.55	0.89 ± 0.50

## Data Availability

The raw data supporting the conclusions of this article will be made available by the authors on request.

## Abbreviations

The following abbreviations are used in this manuscript:

RD	retinal detachment
ARN	acute retinal necrosis
VA	visual acuity
PPV	pars plana vitrectomy
BARN	bilateral ARN
PORN	progressive outer retinal necrosis
VZV	varicella zoster virus
CMV	cytomegalovirus
EBV	Epstein–Barr virus
HSV	herpes simplex virus
NHR	necrotizing herpetic retinopathy
PCR	polymerase chain reaction
PVR	proliferative vitreoretinopathy
BCVA	best corrected visual acuity
